# EEMD-Based Steady-State Indexes and Their Applications to Condition Monitoring and Fault Diagnosis of Railway Axle Bearings

**DOI:** 10.3390/s18030704

**Published:** 2018-02-27

**Authors:** Cai Yi, Dong Wang, Wei Fan, Kwok-Leung Tsui, Jianhui Lin

**Affiliations:** 1 School of Automobile and Transportation, Xihua University, Chengdu 610039, China; 2 Department of Systems Engineering and Engineering Management, City University of Hong Kong, Tat Chee Avenue, Kowloon, Hong Kong 999077, China; weifan8-c@my.cityu.edu.hk (W.F.); kltsui@cityu.edu.hk (K.-L.T.); 3 State Key Laboratory of Traction Power, Southwest Jiaotong University, Chengdu 610031, China; lin13008104673@126.com

**Keywords:** steady-state index, threshold, railway axle bearing fault diagnosis, multiple bearing defects, the coefficient of variation, a shape function

## Abstract

Railway axle bearings are one of the most important components used in vehicles and their failures probably result in unexpected accidents and economic losses. To realize a condition monitoring and fault diagnosis scheme of railway axle bearings, three dimensionless steadiness indexes in a time domain, a frequency domain, and a shape domain are respectively proposed to measure the steady states of bearing vibration signals. Firstly, vibration data collected from some designed experiments are pre-processed by using ensemble empirical mode decomposition (EEMD). Then, the coefficient of variation is introduced to construct two steady-state indexes from pre-processed vibration data in a time domain and a frequency domain, respectively. A shape function is used to construct a steady-state index in a shape domain. At last, to distinguish normal and abnormal bearing health states, some guideline thresholds are proposed. Further, to identify axle bearings with outer race defects, a pin roller defect, a cage defect, and coupling defects, the boundaries of all steadiness indexes are experimentally established. Experimental results showed that the proposed condition monitoring and fault diagnosis scheme is effective in identifying different bearing health conditions.

## 1. Introduction

**Figure 1:**
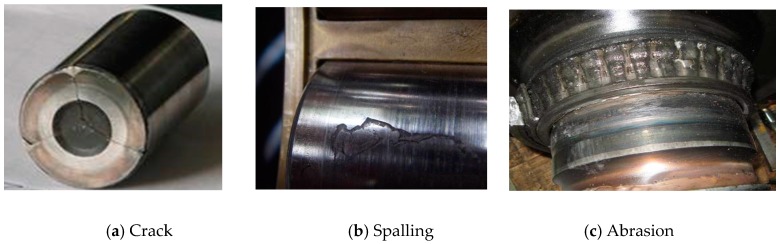
Different railway axle bearing defects.

Although most mechanical systems are protected by vibration shutdown systems, an unexpected shutdown scenario is still not completely avoidable. Mechanical systems normally reach a steady state during their operation. Thus, steadiness is crucial to ensure machine safety. Once the steadiness of a machine and its critical components are quantified, the health states of the machine and its components can be observed indirectly. For example, railway axle bearings are one of the most important components to transmit traction power, bear dynamic radial loads of vehicles and keep the stability and stationariness of train running [1]. An increase of train running speeds makes axle bearings commonly operate under harsh conditions, such as random impacts, natural wear, an exposure to rapid humidity and thermal variations, and rolling contact fatigue. In Figure 1, a long-term alternating stress operating circumstance and heavy loads easily caused axle bearing defects. If an axle bearing defect is not immediately detected, it may lead to damages of other train components even tracks [2]. Consequently, on-line condition monitoring and fault diagnosis of the axle bearing defects are of great importance to avoid train stoppages and even catastrophic derailments.

As stated previously, the importance of stability of railway axle bearings is self-evident. Before steadiness indexes are explored in this paper, it is necessary to review prevalent axle bearing detection technologies and discuss their effectiveness. Railway axle bearing detection has attracted considerable attention with an increase of train speeds, and nowadays many technologies, such as thermal signal detection [3], acoustic emission technology [4], acoustic analysis [5], and vibration acceleration signal analysis [1,6,7,8], have been investigated for this purpose. However, thermal signals may not become evident until a severe bearing fault occurs. How to effectively process acoustic emission signals and interpret their severe attenuations is still challenging. Noises from train traction systems, traction power systems, as well as aerodynamic forces, may contaminate signals acquired by acoustic arrays. Consequently, vibration signals for fault detection of railway axle bearings are concerned a lot in the past years. Vibration signal processing methods in a time-frequency domain have been employed for the axle bearing fault detection. Yi et al. [1] proposed an IMF’s confidence index algorithm extracted from ensemble empirical mode decomposition (EEMD) for faults diagnosis of railway axle bearings. Zhao et al. [6] proposed a fault impulses detection and recovery method via an improved harmonic product spectrum to estimate the defect sizes of train bearings. Cao et al. [7] used empirical wavelet transform for fault diagnosis of wheel bearings. Li et al. [8] proposed a novel feature selection method based on a multi-scale morphological filter to detect train axle bearing faults. Li et al. [9] designed a fault classifier based on multi-feature parameters and a BP neural network to automatically recognize fault patterns of axle box bearings. Besides, more effective techniques based on bearing defect frequency detection are summarized in references [10,11]. These approaches aimed to extract axle bearing defect frequencies so as to identify the abnormal bearing condition and its relevant fault patterns. Nevertheless, a bearing defect frequency $f_{k}$ is varied over time a traction motor frequency $f_{n}$ is fluctuated and changed [12]. Especially, when $f_{k}$ is a variable, it is not easy to use to detect multiple defects.

Unlike the aforementioned axle bearing detection methods, health indicators are capable of estimating the current state of components and systems of interest. An example of directly measurable degradation indicators is the length of a crack in a structure [13,14,15], while examples of a direct measure of bearing faults and their degradation levels are seldom reported [16,17]. Although several health indicators have been developed for fault diagnostics and prognostics of different types of industrial components, solutions are tailored to a component and specific characteristics of some monitored signals [18,19,20,21]. According to the main idea of health indicators, once a set of features are extracted from raw measurements, it is possible to combine them into a single health indicator because individual features may only contain partial and independent information about equipment degradation states. Zhang et al. [22] proposed an approach based on principal component analysis and hidden Markov models to define a health indicator for machine prognostics. Coble et al. [23] defined a health indicator as a linear combination of auto-associative kernel regressions (AAKR) of different signals. The coefficients of the linear combination were obtained by using a genetic algorithm to assess the capability of a health indicator. Baraldi et al. [24] considered various signal extraction techniques, including statistical indicators, empirical mode decomposition (EMD), wavelet transform and Fourier transform to define a health indicator as the residual of the AAKR model for fault diagnostics and prognostics.

The objective of this paper is to propose some steadiness based health indexes for quantitatively and qualitatively estimating axle bearing running health conditions. These indexes not only can be used to characterize the current steady state and detect fault patterns of axle bearings but also can act as a significant base features to construct a systematic health indicator. The proposed indexes differ from the previous statistical parameters in the literature, such as Jensen-Rényi divergence [25], entropy [26], kurtosis [27,28,29], etc. Intrinsic and fundamental attributes of signals are used to construct and represent steadiness. A signal can be considered as a description of a moving process. Various intrinsic properties of the moving process can be utilized but are mostly left untapped. Stability generally refers to the ability of a system to maintain its inherent characteristics as time goes on. Since stability is always relative to time, it is necessary to determine the relationship between the intrinsic characteristics of a system and time. There are two ways to realize this purpose. The first way is to measure the time required to take a specific change with an inherent characteristic, and the second is to measure a variation of an inherent characteristic within a fixed period of time. Therefore, selection and definition of internal parameters are of great concern. Time, speed and space shape are the most important and basic attributes of the movement, and they can well stimulate and embody its interior characteristics. Related to a signal, these attributes can be converted in a time domain (T-domain), an instantaneous frequency domain (IF-domain) and a shape domain (S-domain), respectively. Considering the adaptability of these indexes, ensemble empirical mode decomposition (EEMD) is used for adaptive signal decomposition. The choice of EEMD is because EEMD has high adaptability and is an improvement on mode aliasing [25,30,31]. The main novelties of this paper are summarized as follows. Firstly, being different from traditional approaches, different health states of axle bearings are considered as deviations from the steady state of vibration signals. Secondly, the proposed steady-state indexes can be used as monitoring parameters for on-line condition monitoring and fault detection. Thirdly, intrinsic properties of vibration signals are tapped to construct steady-state indexes. Fourthly, the use of EEMD is taken as an example for signal decomposition. For the applications of our proposed steady-state indexes, we focus on steadiness measures of railway axle bearings. Based on steady-state indexes of railway axle bearings, an on-line condition monitoring and, fault diagnosis scheme of railway axle bearings is realized and thus condition-based maintenance can be timely conducted to prevent any sudden system shutdown caused by bearing failures.

The rest of this paper is organized as follows: the fundamental details of EEMD are briefly introduced in Section 2. The theory and construction of steady-state indexes are proposed in Section 3. The experimental platform is described in Section 4. The proposed indexes are applied to quantify vibration signals of train axle bearings in Section 5. Section 6 discusses results of three steady-state indexes and finally conclusions are drawn.

## 2. Adaptive Signal Decomposition

### 2.1. Ensemble Empirical Mode Decomposition

EMD is an adaptive signal processing method to decompose a nonlinear and non-stationary time series into a set of orthogonal components named as intrinsic mode functions (IMFs) [25]. The remarkable advantage of EMD is high self-adaptability in nature, which means that EMD does not use any predefined basis function. Nevertheless, EMD has a mode aliasing problem, which is alleviated through a process of a noise-assisted data analysis method by adding finite white noises to a raw signal before EMD is conducted. The improved EMD is called EEMD [32]. For a series x(t), the steps of EEMD are introduced as follows:

Step 1: Generate a new series by adding white noises into an original series x(t). When the white noises, which consist of components of different frequency scales and are uniformly distributed with a constant standard deviation, are added to the original signal of concern to form a series y(t) corrupted by the white noises. Here, the white noises will make different scale signals reside in their corresponding IMFs.
Step 2: Identify all local maximum and minimum of the time series y(t).
Step 3: Generate an upper envelope $e_{u}\left(t\right)$ and a lower envelope $e_{l}\left(t\right)$ of y(t).
Step 4: Calculate the mean m(t) from the upper and lower envelopes.
Step 5: Calculate the difference between y(t) and m(t) as the first component h(t).
Step 6: The sifting process has to be iterated several times until h(t) satisfies the definition of an IMF, and then the first IMF $c_{1}$ is generated.
Step 7: A residue generated by subtracting the first IMF from the time series y(t) is treated as a new series, and then repeat Steps 2 to 6 to gain all IMFs and an ultimate residue.

The essence of EEMD is to add different white noise series before EMD is conducted so as to cancel the final mean of some intrinsic mode functions. The finial mean of intrinsic mode functions stays within natural dyadic filter windows and thus significantly reduce mode mixing and preserve a dyadic property [32,33]. It is suggested that the amplitude of added white noises is taken as 0.2 times of the standard deviation of a signal [32]. If an amplitude of added noises is too large, it would result in a large number of redundant IMFs [34]. In contrast, if the amplitude of added noises is too small, no effect on mode mixing prevention happens [35].

According to the above steps, the original signal x(t) is decomposed into n-IMFs using EEMD as follows:

(1) $$x\left(t\right)=\sum_{i=1}^{n}c_{i}\left(t\right)+r_{n}\left(t\right)$$

where $r_{n}\left(t\right)$ is a residual function and $c_{i}\left(t\right),\;n=1,2\ldots ,n$ are the IMFs of different frequency components ranging from high frequencies to low frequencies. Unlike Fourier transform and wavelet transform where a predetermined basis function is required, EEMD fully relies on the characteristics of an original signal.

The IMFs have different energy scales due to the fact that different frequency components resulting from the decomposition process of EEMD [36]. Energies of different IMFs can be used to characterize a signal. The energy of the ith IMF is given as follows:

(2) $$E_{i}=\int_{-\infty }^{\infty }{\left|c_{i}\right|}^{2}dt\;\left(i=1,\;2,\;\cdots \cdots ,\;N\right)$$

where N is a total number of data points in a signal. These estimated energies can be normalized into a set of a probability distribution as follows [25]:

(3) $$p_{i}=\frac{E_{i}}{E},\;\mathrm{where}\;E=\sum_{i=1}^{n}E_{i}\;\Rightarrow \;\sum_{i=1}^{n}p_{i}\;=1$$

where $p_{i}$ is the percent of the energy of the ith IMF $c_{i}\left(t\right)$ to the total signal energy E or a probability of energy $E_{i}$ in the total signal E, for one particular signal.

### 2.2. Selection of IMFs

After EEMD is performed on a signal, a series of IMFs are obtained. Only some selected IMFs are preferable to exhibit bearing fault-related information, and irrelevant IMFs should be excluded in further analyses. Yi et al. [1] proposed an IMF’s confidence index algorithm to select the most sensitive IMFs for bearing fault diagnosis, and a new signal was reconstructed by the selected IMFs. Being different from this study, here a simpler method for selecting IMFs will be proposed to facilitate the development of on-line implementing.

The central frequencies of IMFs will arrange in order from high to low after EEMD is conducted, and each IMF represents a set of frequency cluster of a particular mode. In order to make process briefness and effectiveness, one IMF as a microcosm of an original signal is selected to estimate the steady state of axle bearings. There are three ways to determine an IMF:

(1) Choose a fixed IMF to derive the unsteadiness of a vibration signal of axle bearings, such as the third IMF or the fourth IMF. The basic property variation of a specific IMF, such as amplitude and energy change and so on, may measure the dynamic instability of axle bearings.
(2) Choose an IMF due to a specific frequency component, such as an IMF with a location of a rotating frequency of axle bearings; in this way, a particular frequency cluster does not always exist in a fixed IMF.
(3) Choose an IMF thanks to a specific characteristic, such as an IMF with the largest energy.

Due to the complicated operating environments and variousness of bearing failure modes, vibration characterization of railway axle bearings is time-varying, and any physical content presented in each IMF would change, so the first way related to a fixed IMF is unreasonable. As aforementioned in the introduction, a defect frequency is forced to become variable, and it is challenging to determine the locations of these defect frequencies in IMFs, so the second method is difficult to implement. However, no problems exist in the third method. An IMF with the maximum energy for any signal always represents the mode of the most powerful frequency cluster. Although it may contain more or less useful information, it is undeniable that it always represents the current vibration state of axle bearings at this moment. Therefore, the third method is a good choice.

## 3. The Definition of EEMD Based Steady-State Indexes

As stated in the Introduction, the degree of steadiness should be the most important criterion for machinery health status. In an ideal case, a normal rotating machine rotates at a constant speed and a dynamic response should be kept in a steady state. In an ordinary way, an experienced train operator can perceive the abnormal vibration of an equipment under a vehicle through standing in a carriage just because of having an intimate knowledge of a steady state. Once a fault arises, a response would be generated and then a system would vary with respect to the original stable state. To a certain extent, this system is unstable and a deviation from a stable state occurs. Therefore, if a stable state or the degree of steadiness deviation can be indirectly measured, a system health condition can not only be identified qualitatively but also can be quantitatively described. However, when a system is unhealthy, it is difficult to measure the current steadiness of the system and it is preferable to measure the deviations of the system from its stable state, which is called as system instability. In a real world, due to environments and other reasons, any system will be affected by unstable factors. For example, it is impossible for axle bearings to maintain a steady state because of irregular track excitation, high-speed aerodynamic force, coupling effect of other components and so on. So, it is not reasonable to measure steadiness such a time-varying variable, and an unstable index should be identified to characterize a deviating degree from an origin steady state so as to master the current basic health condition of axle bearings serving in such a complicated system. Here, such instability indexes are called steady state deviators. If a system is assumed to be in a completely stable state, its steady state deviator is zero. When the steady-state deviator is greater than zero, it means that the system deviates from the steady state, and the degree of deviation depends on the magnitude of the steady state deviators.

Although experienced operators do not depend on time-frequency variations to judge the unsteadiness, in order to visualize and quantify this perceptual feeling, it is a visible way to tap a vibration signal itself to dig intrinsic information. Typically, indicator extraction is performed by: (i) pre-processing raw data to reduce measurement and process noises; and (ii) extracting characteristic trends using statistical indicators in a time domain, such as mean and standard deviation, and other indicators in a frequency domain or a time-frequency domain [18,19,20,24]. However, these dimensional indexes have pertinence and they are not conducive to make methods easily adapt to various machines conditions with minimum adjustments. Hence, dimensionless indexes are preferable.

### 3.1. A Steady-State Index in Time Domain

**Figure 2:**
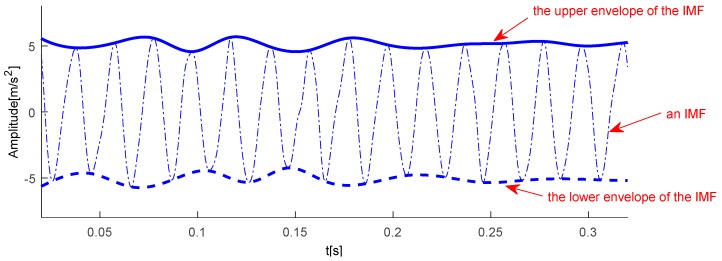
Upper and lower envelopes of an IMF obtained from an axle bearing vibration signal.

When an axle bearing is running at a steady state, its vibrations will fluctuate over time in a small range. The amplitude of a vibration signal is the most prominent property in a time domain because of the emergence of an abnormal shock in an unsteady state. A dispersion will increase with respect to amplitude fluctuation. An IMF with the strongest energy has been determined as a basic reference signal of measurement stability, and amplitude fluctuation of this specific IMF in a time domain is concerned firstly. In the aforementioned EEMD decomposition, the envelope of a signal is plotted in Figure 2. For one IMF, the upper envelope $e_{u}^{i}\left(t\right)$ and lower envelope $e_{l}^{i}\left(t\right)$ of $c^{i}\left(t\right)$ are slightly asymmetric due to the adopted stoppage criteria. Here, the upper envelope is to represent an amplitude variation to simplify a computation process and the linear spline method proposed by Wu and Huang [32] is used to suppress any computational errors caused by the end effect.

The mean and standard deviation of the upper envelope are effective parameters to characterize a variation with respect to another state. However, due to a measurement unit, as mentioned above, these parameters are always restricted by a physical structure and an environmental factor, and they have pertinence and are not conducive to make methods easily adapt for various machine conditions with minimum adjustments. Here, a steady-state index in a time domain is proposed as the ratio of the standard deviation to the mean of the upper envelope curve and it is abbreviated as $SI_{T}$.

Suppose that $\left\{X_{m,1},X_{m,2},\;\cdots ,X_{m,n}\right\}$ are subgroups of size n at time m=1,2,⋯ and that there is independent both within and between these subgroups. Let $\mu_{m}>0$ and $\sigma_{m}$ be the population mean and the standard deviation, respectively, at time m. Assuming that $X_{m,j}\sim N\left(\mu_{m},\sigma_{m}=\gamma_{m}\mu_{m}\right)$, where j=1,2,⋯,n and $\gamma_{m}=\frac{\sigma_{0}}{\mu_{0}}=\gamma_{0}$. Here, $\gamma_{0}$ is the in-control target value of this rate; while $\sigma_{0}$ and $\mu_{0}$ are the in-control mean and standard deviation, respectively. This indicates that the parameters $\mu_{m}$ and $\sigma_{m}$ may change from one subgroup to another although $\gamma_{m}$ must be equal to a predefined value $\gamma_{0}$, which is common to all the subgroups. In the presented case, the distribution of the upper envelope curve $e_{u}^{i,j}$ of the ith IMF is unknown, and the ratio of the standard deviation to the mean is then defined as follows:

(4) $$SSI_{T}=\frac{\sigma_{u}^{i}}{\mu_{u}^{i}}$$

where the upper envelope curve mean $\mu_{u}^{i}$ and its standard deviation $\sigma_{u}^{i}$ are computed as $\mu_{u}^{i}=\sum_{j=1}^{n}e_{u}^{i,j}/n$ and $\sigma_{u}^{i}=\sqrt{\sum_{j=1}^{n}{\left(e_{u}^{i,j}-{\bar{e}}_{u}^{i,j}\right)}^{2}/\left(n-1\right)}$, respectively.

Mathematically, the ratio of the standard deviation to the mean has another nomenclature in probability theory and statistics, namely the coefficient of variation (CV) which is also known as a relative standard deviation and a standardized measure of dispersion of a probability distribution or frequency distribution. CV was widely used in analytical chemistry to express the precision and repeatability of an assay [37], and in biology to assess pain or other something [38]. In addition, CV has been utilized by economists and investors in economic models and in determining the volatility of a security. However, there are few applications in the engineering field, especially for bearing detection and fault diagnosis. Curto and Pinto [39] claimed a zero or negative value leads to a meaningless ratio of CV. Therefore, CV is used to compare the relative variability of a strictly positive random-variable distribution. By definition, the steady-state index with the upper envelope curve is defined in a range (0, +∞).

### 3.2. A Steady-State Index in an Instantaneous Frequency Domain

Frequency domain refers to the analysis of mathematical functions or signals with respect to a frequency rather than time and it is another perspective to explore a vibration signal. Instantaneous frequency (IF) is to understand detailed mechanisms of nonlinear and nonstationary processes, although it has always elicited strong opinions in data analysis and communication engineering communities, covering the range from “banishing it forever from the dictionary of the communication engineer” [39] to being a “conceptual innovation in assigning physical significance to the nonlinearly distorted waveforms” [40]. Historically, IF was computed from an analytic signal through the Hilbert transform. Here, frequency values have to be an instantaneous frequency computed with a zero-crossing method considering only full wavelength cases. The reason is visible that the contribution of nonlinearity from a steadiness index computation in the case of a full instantaneous frequency proposed by Huang et al. [41] must be separated. The most significant contribution is that a new normalization scheme was proposed, which is an empirical amplitude-modulated (AM) and frequency-modulated (FM) decomposition method which enables us to separate any IMF empirically and uniquely into an envelope (AM) and carrier (FM) parts [41]. In this method, the main steps are given as follows:

Step 1: Decompose an original signal x(t) into IMFs using EEMD.
Step 2: Identify all local maxima of the absolute value for the ith IMF $c_{i}\left(t\right)$. Here that the normalized data that are symmetric with respect to a zero axis would be guaranteed by using the absolute value fitting.
Step 3: Constructing a cubic spline curve by connecting all these maxima points. The spline curve $e_{i}\left(t\right)$ is designated as an empirical envelope of the IMF.
Step 4: Normalization of the envelope curve $e_{i}\left(t\right)$ by $f_{i}^{1}\left(t\right)=\frac{c_{i}\left(t\right)}{e_{i}^{1}\left(t\right)}$ with $f_{k}\left(t\right)$ as normalized data.
Step 5: Ideally, $f_{i}^{1}\left(t\right)$ should have all the extrema with unity value. At the locations of fast changing amplitudes, the envelope spline line passing through the maxima can go below some data points. If the normalized data still have amplitudes higher than unity occasionally, unfortunately, the normalization procedure can be implemented repeatedly with $e_{i}^{2}\left(t\right)$ defined as the empiric envelope for $f_{i}^{1}\left(t\right)$, and so on. So if $\left|f_{i}^{1}\left(t\right)\right|<1$, the calculation process will black out; if not, Steps 2 to 4 will be repeated until $f_{i}^{k}\left(t\right)$ satisfies that $\left|f_{i}^{k}\left(t\right)\right|=\left|\frac{f_{i}^{k-1}\left(t\right)}{e_{i}^{k}\left(t\right)}\right|\leq 1$, where k is the number of repeats.

The normalization is complete and it is then designated as the empirical FM part of the IMF, $f_{i}^{k}\left(t\right)=cos\phi_{i}\left(t\right)=F_{i}\left(t\right)$ where $F_{i}\left(t\right)$ is a purely FM function with unity amplitude. It should be noted that the normalization process could cause some deformation of original data, but the amount of deformation is negligible for rigid controlling points for periodicity provided by the zero-crossing points in addition to the extrema. The zero-crossing are totally alternated by the normalization process.

Since the FM component is stationary, the Hilbert transform can be better applied to FM and its expression is given as follows:

(5) $$H\left[F_{i}\left(t\right)\right]=\frac{1}{\pi }P.V.\int_{-\infty }^{\infty }\frac{f_{i}^{k}\left(\tau \right)}{t-\tau }d\tau$$

where $H\left[F_{i}\left(t\right)\right]$ is the Hilbert transform of $F_{i}\left(t\right)$, $P.V.\int_{-\infty }^{\infty }\frac{f_{i}^{k}\left(\tau \right)}{t-\tau }d\tau$ is the Cauchy principal value. Considering the sensitivity of an instantaneous frequency to noises, here a damped instantaneous frequency computing method was adopted which is defined as follows [42]:

(6) $$IF_{i}\left(t\right)=\frac{1}{2\pi }\frac{F_{i}\left(t\right)\frac{dH\left[F_{i}\left(t\right)\right]}{dt}-H\left[F_{i}\left(t\right)\right]\frac{dF_{i}\left(t\right)}{dt}}{s\left(t\right)+\sigma s_{m}\left(t\right)}$$

where $s\left(t\right)=F_{i}{}^{2}\left(t\right)+H^{2}\left[F_{i}\left(t\right)\right]$, $s_{m}\left(t\right)$ is the maxima of s(t), and 0<σ<1 is the damping factor.

**Figure 3:**
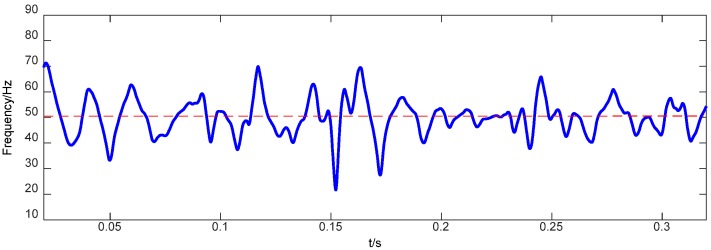
Instantaneous frequency of the IMF corresponding to Figure 2.

Obviously, keeping an FM component constant is the key to the IF computing process essentially. The empirical FM classification can effectively eliminate the influence of amplitude fluctuation on a phase function. According to the aforementioned IF computational procedure, the IF values of the IMF corresponding to Figure 2 can be computed and shown in Figure 3. Here, a bearing rotation speed of 50 Hz is a dominating instantaneous frequency, and it is so accurate that minute inter-wave modulations that could be used as a discriminator for a faulty bearing.

Being similar with the steadiness index definition in a time domain, a new index in a frequency domain is defined as

(7) $$SSI_{F}=\frac{\sigma_{IF}^{i}}{\mu_{IF}^{i}}$$

where the instantaneous frequencies mean $\mu_{IF}^{i}$ and its standard deviation $\sigma_{IF}^{i}$ of the ith IMF are computed as $\mu_{IF}^{i}=\sum_{j=1}^{n}f_{i}^{j}/n$ and $\sigma_{IF}^{i}=\sqrt{\sum_{j=1}^{n}{\left(f_{i}^{j}-\mu_{f_{i}}^{j}\right)}^{2}/\left(n-1\right)}$, respectively.

### 3.3. A Steady-State Index in a Shape Domain

Under ideal conditions, the trajectory of a point motion will follow a certain rule, so it will have a fixed shape pattern. Once a steady departure occurs, the shape pattern will change accordingly. Therefore, the shape pattern is considered as the third perspective to investigate the stability of axle bearing vibration signals. As aforementioned above, any oscillatory sinusoidal wave multiplied by a smooth envelope function will satisfy the definition of IMFs. Many commonly used dictionaries include IMFs. For example, elements in a Gabor dictionary generated by applying a Gaussian envelope on a sinusoidal wave are IMFs. Some wavelets, such as all of the elements of a Fourier dictionary are defined as IMFs, and a Morlet wavelet also satisfies the conditions of IMFs. Inspired by the EMD method, Hou and Shi [43] used a variant of EMD dictionary to construct a sparse decomposition of a signal via a nonlinear optimization and defined a shape function as a generalization of an IMF. For any raw data, x(t), to be expanded in terms of IMFs based on Equation (1) as follows:

(8) $$x\left(t\right)=\overset{N}{\underset{j=1}{\sum }}a_{j}\left(t\right)\cos\theta_{j}\left(t\right)$$

where the amplitude function $a_{j}\left(t\right)$ has to be smoother than the phase function $cos\theta_{j}\left(t\right)$. Then, a more generalized form of the expansion of a signal is defined as in terms of a shape function:

(9) $$x\left(t\right)=\overset{N}{\underset{j=1}{\sum }}a_{j}\left(t\right)S_{j}\left(\theta_{j}\left(t\right)\right)$$

where the shape function $S_{j}\left(\theta_{j}\left(t\right)\right)$ is any 2π period function not necessarily of the cosine form nor smooth [42]. Obviously, this function form is much more general, and it could be used to accommodate many special forms of data such as a delta function or functions with sharp steps or jumps. Here, the shape function can be defined as the phase locked-in mean of the signal. For the shape function existing in Equation (9), data have to be periodic with uniquely defined cycles and phase points. In the present particular application to railway axle bearings, the approximately periodic property of bearings is used to derive the shape function from data, and then compute the steadiness of a vibration signal.

As the definition of the IF for the ith IMF $c_{ix}\left(t\right)$ of an origin signal x(t) with a FM part, the AM part $A_{ix}\left(t\right)$ is defined as follows:

(10) $$A_{ix}\left(t\right)=\frac{c_{ix}\left(t\right)}{F_{ix}\left(t\right)}$$

Therefore, from Equation (10), then an analytic signal is defined as:

(11) $$C_{ix}\left(t\right)=c_{ix}\left(t\right)+iF_{ix}\left(t\right)=A_{ix}\left(t\right)e^{i\theta_{ix}\left(t\right)}$$

where $A_{ix}\left(t\right)$ is the amplitude of $c_{ix}\left(t\right)$ and $\theta_{ix}\left(t\right)$ is the instantaneous phase of $c_{ix}\left(t\right)$.

In a similar way, $C_{jy}\left(t\right)=A_{jy}\left(t\right)e^{i\theta_{jy}\left(t\right)}$ is defined for the jth IMF $c_{jy}\left(t\right)$ for a signal y(t). The signals x(t) and y(t) are called as n:m phase synchronization when the following inequality holds as:

(12) $$\theta_{ix\to jy}\left(t\right)=\left|n\theta_{ix}\left(t\right)-m\theta_{jy}\left(t\right)\right|\leq const$$

where n and m are two positive integers. In the present signal, the 1:1 PS is considered with a periodic property.

A mean phase is commonly used as a measure of phase synchrony, and it is given as follows:

(13) $$\lambda \left(ix,jy\right)=\frac{1}{N}\left|\sum_{l=1}^{N}e^{i\left(\theta_{ix}\left(l\Delta t\right)-\theta_{jy}\left(l\Delta t\right)\right)}\right|$$

where Δt is the sampling interval and N is the length of two signals in samples.

Note that the value of λ is restricted to the interval [0,1]. Here, λ=0 indicates that the IMF $c_{ix}\left(t\right)$ and $c_{jy}\left(t\right)$ are not synchronized at all, while λ=1 implies the phase difference is a constant i.e., perfect phase synchronization.

Here the lock-in method is described as a multiplication of targeted IMFs $C_{i}^{\lambda }\left(x\right)$ with a synchronal phase by a weighting factor K. Usually, the process is called the lock-in correlation procedure [44]. According to the Equations (9)–(13), the shape function $S^{L}$ of axle bearing vibration signals is defined through EEMD for synchronous correlation by the linear averaging over N lock-in periods:

(14) $$S^{L}=\frac{1}{MN}\sum_{i=1}^{M}\sum_{j=1}^{N}K_{j}^{L}C_{i}^{\lambda }\left(t\right)$$

where L is phase position, N is number of periods, M is the number of targeted IMFs, $K_{j}^{0^{{}^{\circ}}}=\cos\left(2\pi \left(j-1\right)\right),\;K_{j}^{90^{{}^{\circ}}}=\sin\left(2\pi \left(j-1\right)\right)$, and $C_{i}^{\lambda }\left(x\right)$ is the analytic signal with a synchronal phase.

**Figure 4:**
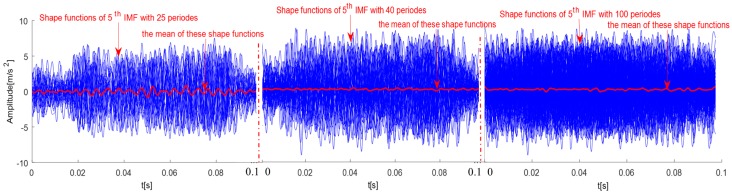
Phase lock in average of one IMF for axle bearing vibration signals.

To achieve this, a unique phase point for implementing the phase locked average must be defined and fixed. There are two possible approaches. The first is to choose a reference point with unique features, such as the absolute maxima in each cycle, but this is not uniquely defined in present data. Due to the interference of external environmental factors, the morphological changes of a vibration signal for axle bearings are significant and disorganized, and it is difficult to locate a fixed reference point. To simplify the computing process, a fixed phase based on the periodicity of a rotation speed is considered. This is a very tempting idea for the rotating speed that is usually fixed and very steady. However, not all the data are strictly periodic. Sometimes, ambient noises and other vibration could contaminate data so much that a clear periodicity may be totally masked. Hence, an alternative approach to the shape function is to apply it to the IMFs, and specifically to the main rotating component. Since a periodic property is extracted with a fixed phase, and such feature makes the average extremely simple. But there is a problem; when the rotating period is not an integer number of data points. Then, the fractional part of the period would accumulate and make the rotational pattern drift. Using the present case shown in Figure 1 as an example, with a fixed period, the phase average is shown in Figure 4. It is interesting that the phase average with the fixed period of a rotation speed results in serious drifting. The more cycles, the more the shape function becomes smooth. Considering the simplicity of the fixed period approach, the fixed period approach should still be considered as an acceptable approximation, provided that it is not implemented on data with a long length. But in order to obtain a stable shape function, the length of data should be long enough to get a stable average. By considering these two conflict requirements, a number around 25 would be a good choice. The length requirement of data makes it feasible to implement the shape function method in real time.

The shape function can be used to evaluate the current operating performance of axle bearings with a healthy one as a reference if it is available. To measure the steadiness, a non-dimensional non-steadiness index based on shape function is defined as:

(15) $$SSI_{S}=\sigma_{\left(S^{L}-S_{0}^{L}\right)}/\sigma_{S_{0}^{L}}$$

in which $\sigma_{S_{0}^{L}}$ is the standard deviation of a shape function for a normal signal, and $\sigma_{\left(S^{L}-S_{0}^{L}\right)}$ is the standard deviation of the residue as the difference of a shape function between detected data and normal data. The larger $SSI_{S}$, the less steadiness an axle bearing system.

## 4. Descriptions of Experiments and Railway Axle Bearing Data

**Figure 5:**
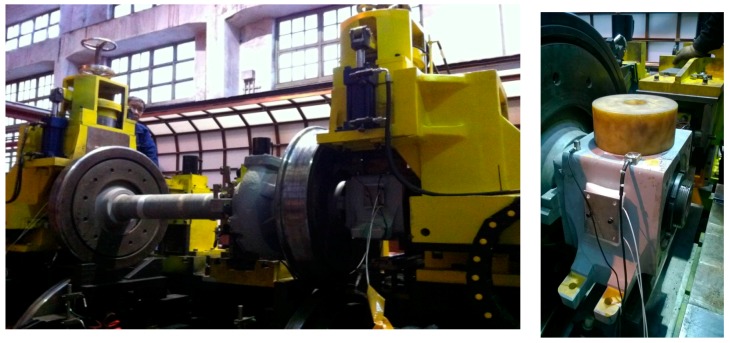
Experimental setup.

In this section, the performance of the proposed steadiness indexes based on EEMD is evaluated by using vibration signals of train axle bearings. The experiments were conducted in a train wheelset test rig displayed in Figure 5. The experimental setup installed on an infinite long rail through double wheel reverse scrolling and different running conditions can be simulated to mimic fault conditions. The wheelset was driven by a hydraulic actuator controlled by a speed controller. A triaxial accelerometer was installed on the edge of a bearing housing to capture vibration signals of axle bearings. In the experiments, a constant load of 2 tons was forced on the surface of an axle box by using a hydraulic cylinder to simulate a servicing condition of axle bearings. Axle bearing vibration signals were collected when a wheelset was running at a speed of 100 km/h which was measured by a shaft encoder. Here, a sampling frequency $f_{s}$ was set to 10,000 Hz and the axle rotating frequency $f_{a}$ was 10.29 Hz.

**Figure 6:**
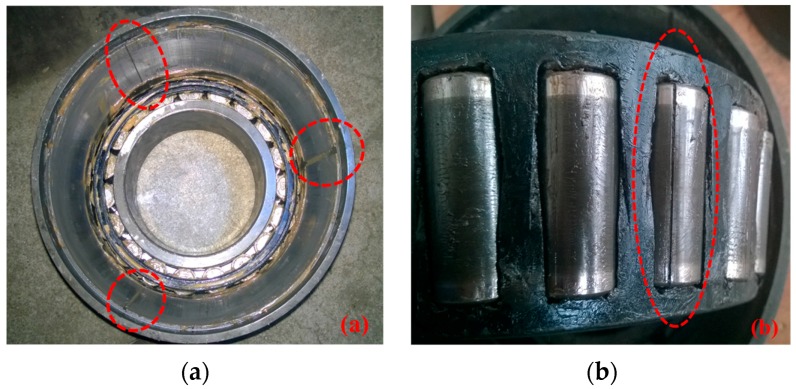

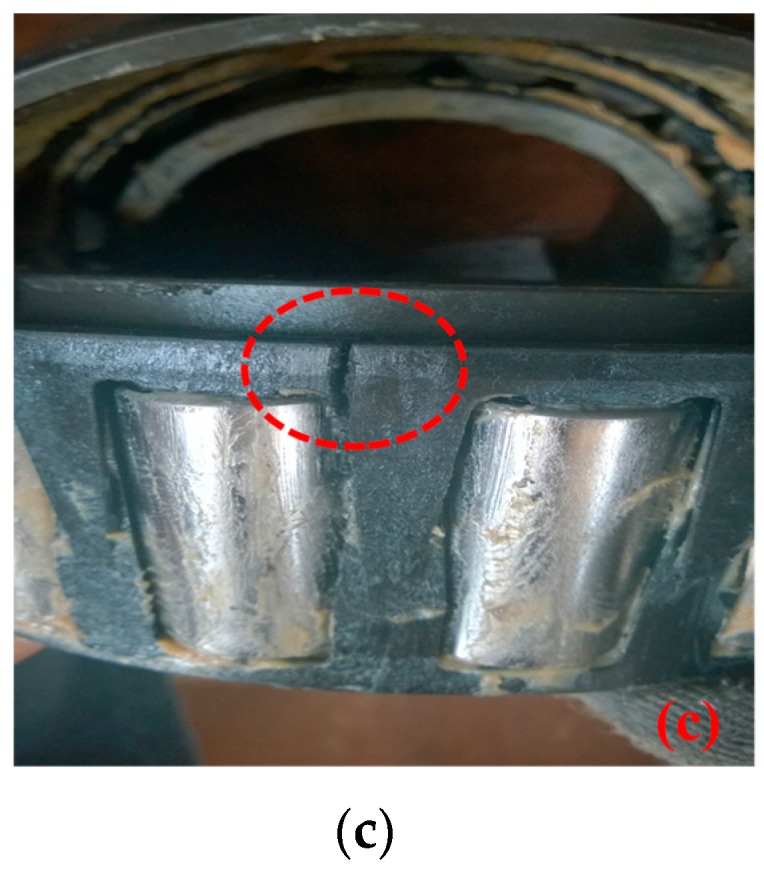
Axle bearing defects: (**a**) three outer race defects; (**b**) a pin roller defect; (**c**) a cage defect.

Double row tapered roller bearings were used in the experiments. The geometric parameters of the axle bearings are given as follows: the roller diameter was 26.9 mm, the pitch diameter was 180 mm, the contact angle was 9°, and the number of rollers was 19. There were three faulty bearings with failures emphasized and re-introduced artificially by a wire-electrode cutting machine based on original faults generated during in-service train. The first bearing had three cracks on an outer race; the second one had a crack on a pin roller; and the last one had a crack in a cage, which are shown in Figure 6a–c, respectively. Each of the outer race defects had a crack length of 20 mm, a crack width of 1 mm and a crack depth of 1 mm. For the pin roller defect, the fault length, width, and depth were 25 mm, 1 mm, and 1 mm, respectively. For the cage defect, the length, width, and depth were 4 mm, 2 mm, and 4 mm, respectively.

According to these different defects, five experiments were designed. In the first experiment, there were no defects on a bearing, which aimed to simulate normal conditions. In the second experiment, only three outer race defects were considered. In the third experiment, only the pin roller defect was considered. In the fourth experiment, only the cage defect was considered. In the fifth experiment, all the outer race, pin roller and cage defects were considered. Even though the three outer race defects were found in a bearing, their defect frequency is still $f_{0}$ rather than $3f_{0}$. This is because a measured vibration signal was the sum of induced vibrations from each of the three defects [45]. Experiments 2, 3 and 4 are relevant to a single type of bearing defects, while experiment 5 is relevant to a multiple type of bearing defects.

**Figure 7:**
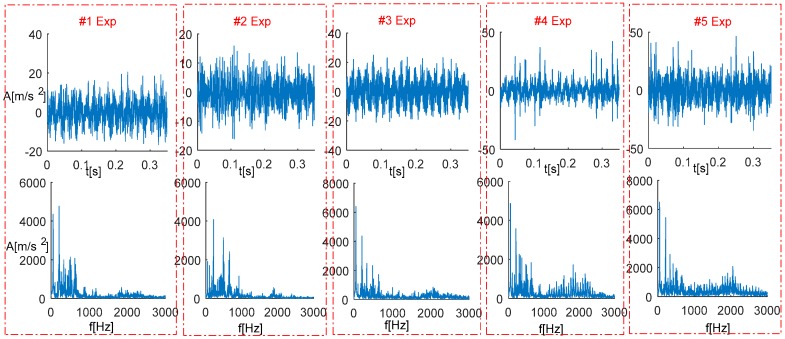
Vibration signals and their frequency spectra from experiments 1 to 5 (#1: a normal bearing; #2: a bearing with outer race defects; #3: a bearing with a pin roller defect; #4: a bearing with a cage defect; #5: a bearing with three kinds of coupling defects).

Vibration signals from a normal bearing and other four faulty bearings, and their corresponding frequency spectra are respectively plotted in Figure 7 where the data series with time changing are shown in the upper row, and their corresponding frequency spectra via Fourier transform are shown in the lower row. It is noted that an axle rotating frequency and its harmonics can be detected while bearing defect frequencies are not identified easily.

**Figure 8:**
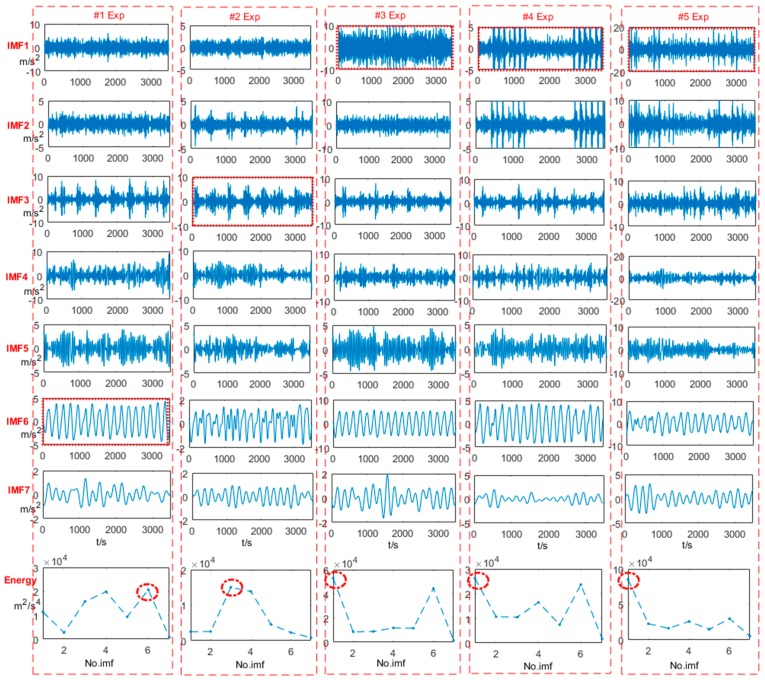
IMFs of vibration signals in Figure 7 obtained by using EEMD and their energy distributions (#1: a normal bearing; #2: a bearing with outer race defects; #3: a bearing with a pin roller defect; #4: a bearing with a cage defect; #5: a bearing with three kinds of coupling defects).

Figure 8 shows that the 1st~7th IMFs of the vibration signals in Figure 7 using EEMD and their energy distributions. Although the same length of data points for axle bearings is used in different conditions, IMFs with the same order are diverse. The bottom row shows the energy distributions of IMFs. The IMFs with the largest energy for each column are selected. For Experiments #1–2, the 6th and 3rd IMFs are the most energetic, respectively. The 1st IMF contains the largest energy for Experiments #3–5. These selected IMFs will be utilized to further characterize the stability of the corresponding vibration signals.

## 5. Application of Steady-State Indexes to Characterizing Railway Axle Bearing States

### 5.1. Characterization of Bearing Vibration Signals Using the SSI in a Time Domain

The axle bearing vibration signals shown in Figure 7 are utilized to illustrate the analysis procedure of the SSI in a time domain. The resulting values of the $SSI_{T}$ of these selected IMFs in Figure 8 are given in Table 1. It is clear that the faulty bearings have the greater $SSI_{T}$ values and show much more unsteady than the normal one. It should be noted that the vibration signal generated from the bearing with three types of coupling defects has the largest $SSI_{T}$ value, and it may be consistent with the commonsense that the vibration signal generated from the multiple coupling defects is more instable than the condition of a single defect. The bearing outer race defect presents the second serious instability; the bearing vibration signal generated from the pin roller defect is the third instability state; and the vibration signal generated from the cage defect is in a relatively stable state compared with the others.

**Figure 9:**
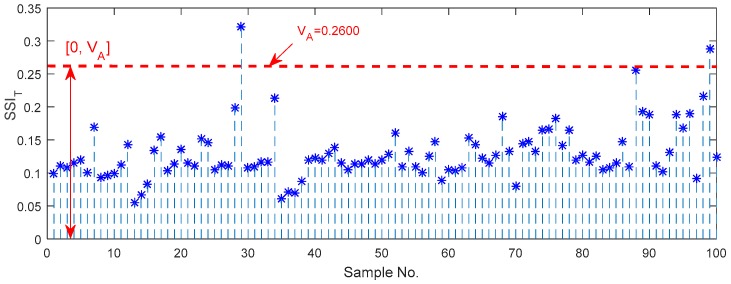
Upper and lower boundaries of the $SSI_{T}$ for quantification of normal axle bearing vibration signals.

The identification of the normal and abnormal states is the first step for the long-term monitoring of railway axle bearings. Hence, the stability boundary of the normal bearing can be considered as a guideline threshold to set up an axle bearing monitoring criterion. A total of 100 vibration samples for the normal bearing are utilized to compute the statistical values of the steady-state index in a time domain, and the results are shown in Figure 9, which satisfies the normal distribution tested by Lilliefores test method. In the empirical sciences, the so-called three-sigma rule of thumb expresses a conventional heuristic that “nearly all” values are taken to lie within the three standard deviations of the mean, i.e., that it is empirically useful to treat 99.7% probability as “near certainty”. Here the three-sigma is used to determine the upper boundary of the $SSI_{T}$. According to the definition of the steady state deviation, the greater the $SSI_{T}$ value, the more unstable the bearing system is. Hence, in this case, the $SSI_{T}$ boundary is $\left[0,V_{A}\right]$, where $V_{A}$ is 0.2600 shown in Figure 9. If the $SSI_{T}$ value is out of the boundary, which indicates the axle bearing is in an abnormal state.

**Figure 10:**
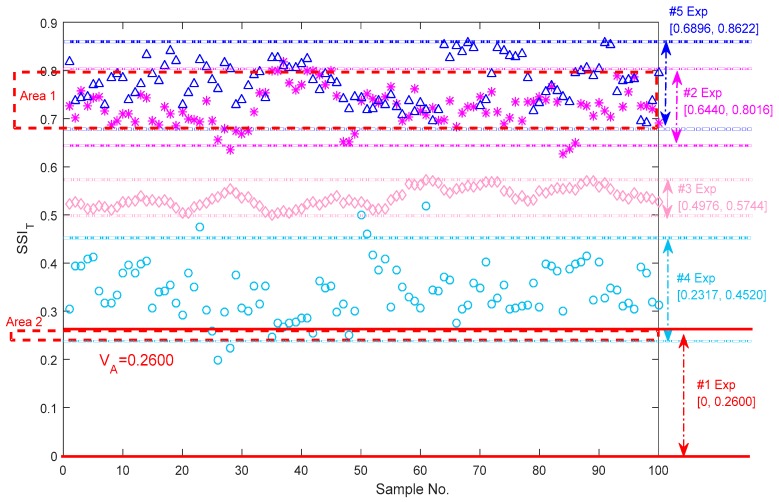
Upper and lower boundaries of the $SSI_{T}$ for distinguishing bearing vibration signals generated from different faults.

Fault diagnosis is always required to identify a specific fault. The statistical values of the other experimental vibration signals are computed by using the same procedure shown in Figure 10. Different colors and icons present all the results. The dotted lines with spherical colors are the upper and lower boundaries for each type of bearing fault and the two thicker red lines present the upper and lower boundaries for the normal axle bearing. It is visible that all the $SSI_{T}$ values for each type of bearing state have a statistical pattern and scatter in a specific value band. Here, the 2-sigma rule, 95.45% of the values lie within the two standard deviation of the mean, is introduced to computing the boundaries of the bearing states relevant to different faults, which is different from the computing rule of making a distinction between the normal and abnormal bearings. The explanations for this reason are made in the following.

The first reason is that a more conservative threshold is needed to distinguish the abnormal state from the normal state. On the other hand, the 2-sigma rule are more common applying in engineering, and a less harsh criterion acts artfully and accurately to distinguish the bearing states relevant to different faults and avoid some overlapped observation regions. It is noted that almost all the results of the abnormal bearings with different faults are beyond the upper boundary $V_{A}$ of the normal bearing except the values located in Area 2, indicating that the threshold has a certain reference value. For the outer race defect bearing vibration signals, the upper and lower boundaries of the $SSI_{T}$ is [0.6440, 0.8016] and it is greater than the [0.4976, 0.5744] and [0.2317, 0.4520] which correspond to the $SSI_{T}$ boundaries of the roller pin defect and the cage defect, respectively. All three cases are the single type of fault, and obvious separating lines of the $SSI_{T}$ between these vibration signals are observed. [0.6898, 0.8622] is the upper and lower boundaries of the $SSI_{T}$ in the case of multiple coupling defects. Although the upper boundary of the $SSI_{T}$ is highest than others, most of the region overlaps with the outer race defect region marked as Area 1 in Figure 10. Hence, it is difficult to divide the outer race defect bearings from the multiple defects coupling bearings.

### 5.2. Characterization of Bearing Vibration Signals Using SSI in Frequency Domain

**Figure 11:**
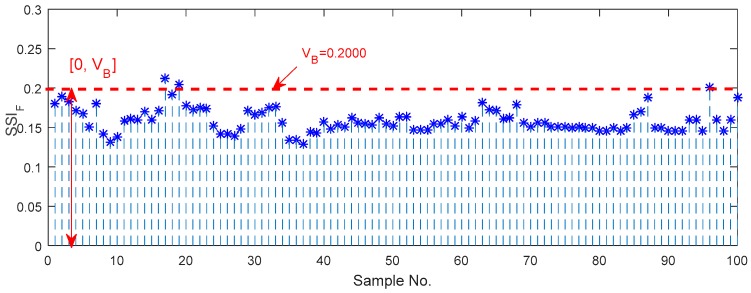
Upper and lower boundaries of the $SSI_{F}$ for characterizing normal axle bearing vibration signals.

The same data samples are utilized to investigate the $SSI_{F}$ for characterization of bearing vibration signals in a frequency domain. Figure 11 shows the upper and lower boundaries of the $SSI_{F}$ for the normal axle bearing, and the guideline threshold is $\left[0,{\;V}_{B}\right]$, where $V_{B}$ is 0.2000. Hence, the resulting value of the $SSI_{F}$ falls outside the boundary band, meaning that it is great than $V_{B}$, and the corresponding bearing is abnormal.

**Figure 12:**
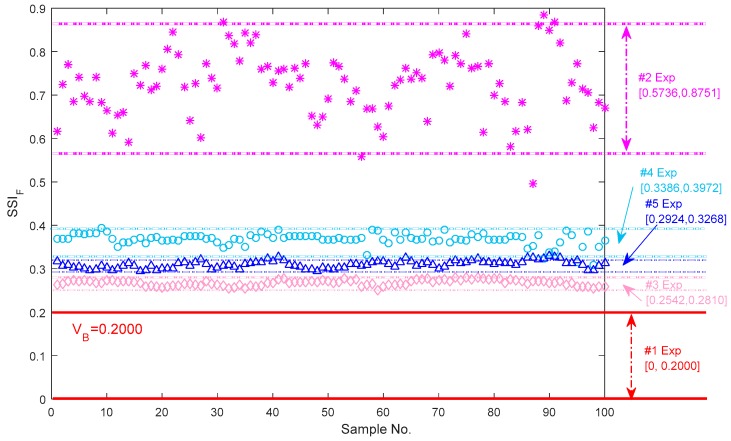
Upper and lower boundaries of $SSI_{F}$ for quantification of bearing vibration signals with the different faults.

The validity of the guideline threshold of $SSI_{F}$ is shown in Figure 12. All the $SSI_{F}$ values of the vibration signals for the bearings with different faults are beyond the boundary scope computed from the normal condition. The $SSI_{F}$ boundary scopes of the axle bearings from the abnormal conditions with the different faults are shown in Figure 12. It is visible that the bearings with the single faults and the coupling faults are separated by the index values clearly. [0.5736, 0.8751], [0.2542, 0.2810], [0.3386, 0.3972] and [0.2924, 0.3268] are the characterization bands for the bearings with the outer race defects, the pin roller defect, the cage defect and the three coupling defects, respectively. It is shown that the frequency steadiness index can well indicate the state of the bearings, and these different values areas can characterize different faults of the axle bearings. It is worth noting that the bearing characterization boundaries if the pin roller defect and the cage defect, and even the coupling defects, demonstrate more narrow bands, and all the distributions of the resulting values are concentrated. However, the distribution of $SSI_{F}$ values for the bearing vibration signal with the outer race defect are dispersed. It is interesting that not only the numerical size, but the sorting of the representation regions are different from the steady-state index $SSI_{T}$ in the time domain. The $SSI_{T}$ of the outer race defect bearing presents the maximum value band than the multiple coupling defects.

### 5.3. Characterization of Bearing Vibration Signals Using the SSI in a Shape Domain

The same 100 × 5 data samples are utilized to investigate the $SSI_{S}$ characterization of bearing vibration signals in a shape domain. As aforementioned in Section 3.3, facing with the two conflict requirements that are a stable shape function with data with a sufficiently long length and the reduction of the phase drifting with short data cycles, a period number of 25 is suggested to compute the shape function. The shape function can be used to evaluate the current performance of an axle bearing with a health condition as a reference via dividing the residue standard deviation for detected signals shape function minus a basic one by the standard deviation of the basic shape function for the health condition, and the non-dimensional non-steadiness index in the shape domain is in view of a basic shape function deriving from the bearing normal condition vibration signal as shown in Equation (15).

**Figure 13:**
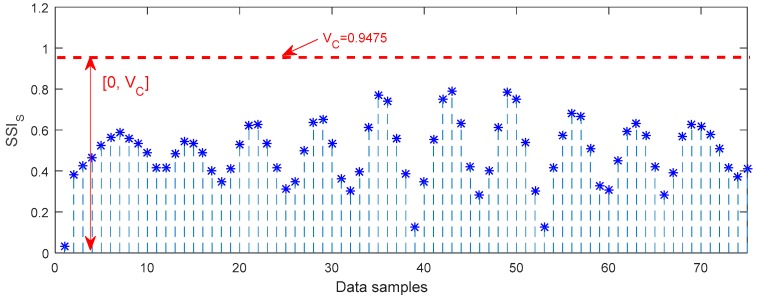
Upper and lower boundaries of the $SSI_{S}$ for characterization of the normal axle bearing vibration signal.

Figure 13 shows the upper and lower boundaries of $SSI_{S}$ for the normal axle bearing, and the guideline threshold is $\left[0,\;V_{C}\right]$, where $V_{C}$ is 0.9475. The larger the value of $SSI_{S}$, the less steadiness the axle bearing system. Hence, the resulting value of $SSI_{S}$ falls outside the boundary band, meaning that it is great than $V_{C}$, and the corresponding bearing is abnormal.

**Figure 14:**
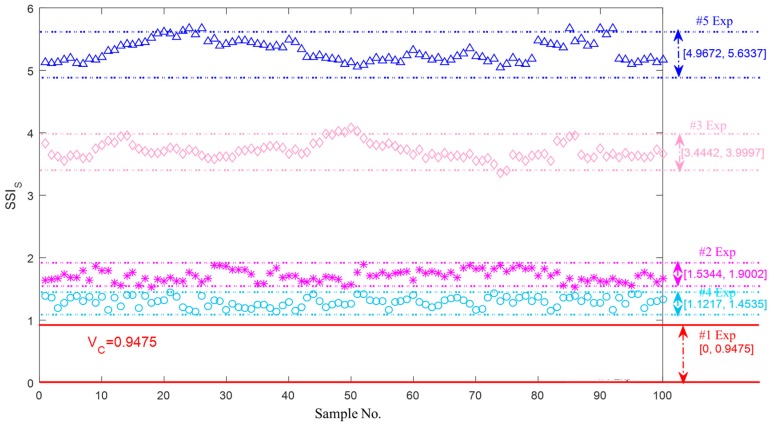
Upper and lower boundaries of $SSI_{S}$ for quantification of the bearing vibration signals with the different faults.

Figure 14 shows the upper and lower boundaries of $SSI_{S}$ for the axle bearing vibration signals with different faults, and it is visible that the classification results are as good as the steadiness index in the frequency domain shown in Figure 12. [0, 0.9475] is the reference value band of the axle bearing vibration signal in a normal condition, and all the others results are beyond the upper boundary. It is thus clear that $V_{C}$ is another effective monitoring guideline threshold. [1.1217, 1.4535], [1.5344, 1.9002], [3.4442, 3.9997] and [4.9672, 5.6337] are the metrological boundaries of $SSI_{S}$ for the cage defect, the outer race defects, the pin roller defect and the coupling multiple defects, respectively. It is noted that the $SSI_{S}$ values of the multiple coupling defects are greater than other abnormal conditions. This conclusion is homologous with the results of the steady-state index in the time domain.

### 5.4. Summary

The contents reported in Section 5.1, Section 5.2 and Section 5.3 are summarized as follows:

(1) All three types of steady-state indexes based on EEMD can distinguish the normal and abnormal axle bearings, and their upper and lower boundaries can be considered as some monitoring guideline thresholds.
(2) All three types of steady-state indexes can be used to classify and characterize the single defects well, and the metrological boundaries for the three types of the single faults are established.
(3) The steady-state indexes in the frequency domain and the shape domain outperform that defined in the time domain for characterizing the multiple coupling defects because the $SSI_{T}$ has some overlapped regions when it is used to distinguish the coupling defects with the outer race defect.
(4) The steady-state index in the time domain has fastest computational efficiency than those defined in the frequency domain and the shape domain. The $SSI_{F}$ needs the extensive time because of the Hilbert transform process. For the steady-state index in the shape domain, the shape function is computed firstly and then the $SSI_{S}$ can be obtained based on two sets of shape functions. But the computational efficiency of the whole computing program is slightly higher than that required in the frequency domain.
(5) The steady-state index in the shape domain is derived from the basic shape function in the normal condition and the shape function related to the detected signal, and a fixed period facilitates the calculation process, but the prior data mode and the fixed period make this method difficult to be implemented. The steady-state index in the time domain has a simplest calculation process, and the $SSI_{F}$ method is in a medium level of difficulty in implementing.
(6) All of the three types of steadiness indexes are non-dimensional so that the different bearing conditions can be characterized by the numerical values in the same domain. However, the threshold values and metrological boundaries between the three index definitions are different from each other. This is because these steady-state indexes do not have a direct mapping with mechanical physical stability. Moreover, these non-steadiness indices are based on different computing principles so that the resulting values are far apart among the three characterization methods.

The performance comparisons of the three types of steady-state indexes are summarized in Table 2.

## 6. Conclusions

In this paper, three EEMD-based steady-state indexes were proposed to characterize the railway axle bearing steadiness states and detect the different defects including the outer race defects, the pin roller defect, the cage defect and the coupling defects. Our findings are summarized as follows:

Firstly, in the experiments, the thresholds for distinguishing the normal and abnormal health states in the time, frequency and shape domains are 0.2600, 0.2000 and 0.9475, respectively. The experimental boundaries in the time domain for the identification of the different bearing defects including the outer race defects, the pin roller defect, the cage defect and the coupling defects are respectively equal to [0.6440, 0.8016], [0.4976, 0.5744], [0.2317, 0.4520] and [0.6898, 0.8622]. Similarly, the experimental boundaries in the frequency domain are respectively equal to [0.5736, 0.8751], [0.2542, 0.2810], [0.3386, 0.3972] and [0.2924, 0.3268]; and the experimental boundaries in the shape domain are respectively equal to [1.1217, 1.4535], [1.5344, 1.9002], [3.4442, 3.9997] and [4.9672, 5.6337].

Secondly, the proposed dimensionless indexes only need a few data and they are independent of prior knowledge about the bearing running environments. Therefore, it is possible to use the proposed indexes to realize on-line condition monitoring and fault diagnosis of railway axle bearings.

## Figures and Tables

**Table 1:** $SSI_{T}$ values of the vibration signals shown in Figure 7.

Experiment No.	#1	#2	#3	#4	#5
Values	0.0992	0.7271	0.5228	0.3038	0.8176

**Table 2:** Comparisons of the performances among steady-state indexes in the time, frequency, shape domains.

Steady-State Indexes	$SSI_{T}$	$SSI_{F}$	$SSI_{S}$
Abnormal diagnosis accuracy	High	High	High
Single defect characterization performance	High	High	High
Coupling defects characterization performance	Low	High	High
Computational efficiency	High	Low	Medium
Difficulty in implementing	Low	Medium	High
